# Learning probabilistic models of hydrogen bond stability from molecular dynamics simulation trajectories

**DOI:** 10.1186/1471-2105-12-S1-S34

**Published:** 2011-02-15

**Authors:** Igor Chikalov, Peggy Yao, Mikhail Moshkov, Jean-Claude Latombe

**Affiliations:** 1 Mathematical and Computer Sciences & Engineering Division, King Abdullah University of Science and Technology, Thuwal 23955-6900, Saudi Arabia; 2 Biomedical Informatics, Stanford University, Stanford, CA 94305, USA; 3 Computer Science Department, Stanford University, Stanford, CA 94305, USA

## Abstract

**Background:**

Hydrogen bonds (H-bonds) play a key role in both the formation and stabilization of protein structures. They form and break while a protein deforms, for instance during the transition from a non-functional to a functional state. The intrinsic strength of an individual H-bond has been studied from an energetic viewpoint, but energy alone may not be a very good predictor.

**Methods:**

This paper describes inductive learning methods to train protein-independent probabilistic models of H-bond stability from molecular dynamics (MD) simulation trajectories of various proteins. The training data contains 32 input attributes (*predictors*) that describe an H-bond and its local environment in a conformation *c* and the output attribute is the probability that the H-bond will be present in an arbitrary conformation of this protein achievable from *c* within a time duration Δ. We model dependence of the output variable on the predictors by a regression tree.

**Results:**

Several models are built using 6 MD simulation trajectories containing over 4000 distinct H-bonds (millions of occurrences). Experimental results demonstrate that such models can predict H-bond stability quite well. They perform roughly 20% better than models based on H-bond energy alone. In addition, they can accurately identify a large fraction of the least stable H-bonds in a conformation. In most tests, about 80% of the 10% H-bonds predicted as the least stable are actually among the 10% truly least stable. The important attributes identified during the tree construction are consistent with previous findings.

**Conclusions:**

We use inductive learning methods to build protein-independent probabilistic models to study H-bond stability, and demonstrate that the models perform better than H-bond energy alone.

## Background

A protein is a long sequence of amino-acids, called residues. Under normal physiological conditions, various forces (electrostatic, van der Waals, ...) lead the protein to fold into a compact structure made of secondary structure elements, α-helices and β-strands, connected by bends (called loops). An H-bond corresponds to the attractive electrostatic interaction between a covalent pair D—H of atoms, in which the hydrogen atom H is bonded to a more electronegative donor atom D, and an electronegative acceptor atom A. Due to their strong directional character, short distance ranges, and large number in folded proteins, H-bonds play a key role in both the formation and stabilization of protein structures [1-3]. While H-bonds involving atoms from close residues along the main-chain sequence stabilizes secondary structure elements, H-bonds between atoms in distant residues stabilize the overall 3D arrangement of secondary structure elements and loops.

H-bonds form and break while the conformation of a protein deforms. For instance, the transition of a folded protein from a non-functional state into a functional (e.g., binding) state may require some H-bonds to break and others to form [4]. So, to better understand the possible deformation of a folded protein, it is desirable to create a reliable model of H-bond stability. Such a model makes it possible to identify rigid groups of atoms in a given protein conformation and determine the remaining degrees of freedom of the structure [7]. Since most H-bonds in a protein conformation are quite stable, it is crucial that the model precisely identifies the least stable bonds. The intrinsic strength of an individual H-bond has been studied before from an energetic viewpoint [5,6]. However, potential energy alone may not be a very good predictor of H-bond stability. Other local interactions may reinforce or weaken an H-bond.

## Methods

### I. Problem statement

Let *c* be the conformation of a protein *P* at some time considered (with no loss of generality) to be 0 and *H* be an H-bond present in *c*. Let *M* (*c*) be the set of all physically possible trajectories of *P* passing through *c* and *π* be the probability distribution over this set. We define the *stability* of *H* in *c* over the time interval Δ by:(1)

where *I* (*q*, *H*, *t*) is a Boolean function that takes value 1 if *H* is present in the conformation *q*(*t*) at time *t* along trajectory *q*, and 0 otherwise. The value  can be interpreted as the probability that *H* will be present in the conformation of *P* at any specified time *t* ∈ (0, Δ), given that *P* is at conformation *c* at time 0. Our goal is to design a method for generating good approximations *σ* of . We also want these approximations to be protein-independent.

### II. General approach

We use machine learning methods to train a stability model *σ* from a given set *Q* of MD simulation trajectories. Each trajectory *q* ∈ *Q* is a sequence of conformations of a protein. These conformations are reached at times *t_i_* = *i* × *δ*, *i* = 0, 1, 2, …, called *ticks*, where *δ* is typically on the order of picoseconds. We detect the H-bonds present in each conformation *q*(*t_i_*) using the geometric criteria given in [8]. Note that an H-bond in a given protein is uniquely identified (across different conformations) by its donor, acceptor, and the hydrogen atom. So, we call the presence of a specific H-bond *H* in a conformation *q*(*t_i_*) an *occurrence* of *H*, denoted by *h*.

For each *h*, we compute a fixed list of predictors, some *numerical*, others *categorical*. Some are time-invariant, like the number of residues along the main-chain between the donor and acceptor atoms. Others are time-dependent. Among them, some describe the geometry of *h*, e.g., the distance between the hydrogen and the donor. Others describe the local environment of *h*, e.g., the number of other H-bonds within a certain distance from the mid-point of H.

We train *σ* as a function of these predictors. The predictor list defines a predictor space ∑ and every H-bond occurrence maps to a point in ∑. Given the input set *Q* of trajectories, we build a data table in which each row corresponds to an occurrence *h* of an H-bond present in a conformation *q*(*t_i_*) contained in *Q*. So, many rows may correspond to the same H-bond at different ticks. In our experiments, a typical data table contains several hundred thousand rows. Each column, except the last one, corresponds to a predictor *p* and the entry (*h*, *p*) of the table is the value of *p* for *h*. The entry in the last column is the measured stability *y* of *h*. More precisely, let *H* be the H-bond of which *h* is an occurrence. Let *l* = Δ/*δ*, where Δ is the duration over which we wish to predict the stability of *h*, and *m* ≤ *l* be the number of ticks *t_k_*, *k* = *i* + 1, *i* + 2,…,*i* + *l*, such that *H* is present in *q*(*t_k_*). The measured stability *y* of *h* is the ratio *m*/*l*. We chose *l* = 50 in most of the tests reported below, as this value both provides a ratio *m*/*l* large enough for the measured stability to be statistically meaningful, and corresponds to an interesting prediction timescale (50ps). Typically, most H-bond occurrences are quite stable: over 25% have measured stability 1, about 50% higher than 0.8, and only 15% less than 0.3.

We build σ as a binary regression tree [9]. This machine learning approach has been one of the most successful in practice. Regression trees are often simple to interpret. The method can work with both categorical and numerical predictors in a unified way, as shown in Section III. Each non-leaf node in a regression tree is a Boolean *split*. So, each node *N* of the tree determines a region of ∑ in which all the splits associated with the arcs connecting the root of the tree to *N* are satisfied. We say that an H-bond occurrence falls into *N* if it is contained in this region. The predicted stability value stored at a leaf node *L* is the average of the measured stability values by all the H-bond occurrences in the training data table that fall into *L*. We expect this average, which is taken over many pieces of trajectories, to approximate well the average defined in Equation (1).

Once a regression tree has been generated, it is used as follows. Given an H-bond *H* in an arbitrary conformation *c* of an arbitrary protein, the leaf node *L* of the tree into which *H* falls is identified by calculating the values of the necessary predictors for *H* in *c*. The predicted stability value stored at *L* is returned.

### III. Training algorithm

We construct a model *σ* as a binary regression tree using the CART method [9]. The tree is generated recursively in a top-down fashion. When a new node *N* is created, it is inserted as a leaf of the tree if a predefined depth has been reached or if the number of *h* falling into *N* is smaller than a predefined threshold. Otherwise, *N* is added as an intermediate node, its split is computed, and its left and right children are created. A split *s* is defined by a pair (*p*, *r*), where *p* is the *split predictor* and *r* is the *split value*. If *p* is a numerical predictor, then *r* is a threshold on *p*, and *s* ≜ *p* <*r*. If *p* is a categorical predictor, then *r* is a subset of categories, and *s* ≜ *p* ∈ *r*. We define the *score w*(*p*, *r*) of split *s* = (*p*, *r*) at a node *N* as the reduction of variance in measured stability that results from *s*. The algorithm chooses the split—both the predictor and the split value—that has the largest score. Only a relatively small subset of predictors selected by the training algorithm is eventually used in a regression tree.

To prevent model overfitting, we limit tree depth to 5 in most of our experiments and limit the minimal number of training samples in an intermediate node to be 10. We further prune the obtained tree using the following adaptive algorithm. We initially set aside a fraction of the training data table called *validation subset*. Once a tree has been constructed pruning is an iterative process. At each step, one intermediate node *N* whose split has minimal score becomes a leaf node by removing the sub-tree rooted at *N*. This process creates a sequence of trees with decreasing numbers of nodes. We compute the mean square error of the predictions made by each tree on the validation subset. The tree with the smallest error is selected.

## Results

### I. Experimental setup

We used 6 MD simulation trajectories picked from different sources and called hereafter *1c9oA*, *1e85A*, *1g9oA_1*, and *1g9oA_2* from [10], *complex* from [11], and *1eia* (generated by us). In all of them the time interval *δ* between two successive ticks is 1ps. Table 1 indicates the protein simulated in each trajectory, its number of residues, the force field used by the simulator, and the duration of the trajectory. Each trajectory starts from a folded conformation resolved by X-ray crystallography.

**Table 1:** Characteristics of the MD simulation trajectories used to create the 6 datasets

Trajectory	Protein	# res.	Force field	Duration	# H-bonds	# occurrences
***1c9oA***	Cold shock protein	66	ENCAD [12] with F3C explicit water model	10ns	263	363463
***1e85A***	Cytochrome C	124	Same as above	10ns	525	1253879
***1g9oA_1***	PDZ1 domain of human Na(+)/H(+) exchanger regulatory factor	91	Same as above	10ns	374	558761
***1g9oA_2***	Same as above	91	Same as above	10ns	397	544491
***complex***	Efb-C/C3d complex formed by the C3d domain of human Complement Component C3 and one of its bacterial inhibitors	362	Amber 2003 with implicit solvent using the General Born solvation method [13]	2ns	1825	348943
***1eia***	EIAV capsid protein P26	207	Amber 2003 with SPC/E water model	2ns	757	379573

From each trajectory we derived a separate data table in which the rows represent H-bond occurrences. Last two columns in Table 1 list the number of distinct H-bonds detected in each trajectory and the total number of H-bond occurrences extracted. Note that *complex* was generated for a complex of two molecules. All H-bonds occurring in this complex are taken into account in the corresponding data table.

The values of the time-varying predictors are subject to thermal noise. Since a model *σ* will in general be used to predict H-bond stability in a protein conformation sampled using a kinematic model ignoring thermal noise (*e.g.*, by sampling the dihedral angles *ϕ*, *ψ*, and *χ*) [7], we chose to average the values of these predictors over *l*' ticks to remove thermal noise. More precisely, the value of a predictor stored in the row of the data table corresponding to an H-bond occurrence in *q*(*t_i_*) is the average value of this predictor in , where . Our analysis shows that *l*' = 50 is near optimal.

The performance of a regression model can be measured by the root mean square error (RMSE) of the predictions on a test dataset. For a data table *T* = {(*x*_1_, *y*_1_), (*x*_2_, *y*_2_)},…, (*x_n_*, *y_n_*)}, where each *x_i_*, *i* = 1,…,*n*, denotes a vector of predictor values for an H-bond occurrence and *y_i_* is the measured stability of the H-bond, and a model *σ*, the RMSE is defined by: . As RMSE depends not only on the accuracy of σ, but also on the table *T*, some normalization is necessary to compare results on different tables. So, in our tests we compute the decrease of RMSE relative to a base model *σ*_0_. The *relative base error decrease* (or *RBED*) is then defined by:  In most cases, *σ*_0_ is simply defined by , *i.e.*, the average measured stability of all H-bond occurrences in the dataset. In other cases, *σ*_0_ is a model based on the H-bond energy.

### II Generality of models trained on multiple trajectories

**Figure 1:**
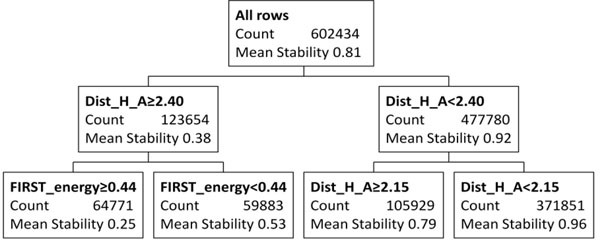
Top 2 layers of a regression tree trained with combination of all tables, except *1c9oA*. The actual tree contains 55 nodes. Each path from the root to a node defines a conjunction of criteria for H-bonds with a certain mean stability. Here, Dist_H_A (the distance between the hydrogen and the acceptor atoms) is the most differentiating predictor. For H-bonds with Dist_H_A≥2.40Å, the mean stability is only 0.38, but it increases to 0.92 if Dist_H_A<2.40Å.

Our goal is to train models to predict the stability of H-bonds in any protein. So, we trained models on data tables obtained by mixing subsets of 5 data tables and we tested these models on the remaining data table. For each combination of 5 data tables, we trained 4 groups of models varying in the tree’s maximal depth (5 or 15) and in the fraction of H-bond occurrences randomly taken from each data table (10% or 50%). For each group we trained 10 models. Hence, in total, 240 models were generated. Table 2 shows the mean RBED value for each combination of data tables and each group of models. In columns 3 through 8 we indicate the data table used for testing the models trained on a combination of the 5 other data tables. Figure 1 shows a partial tree trained with combinations of all tables, except *1c9oA*.

**Table 2:** Mean RBED values of models trained on multiple trajectories

Fraction of data	Max tree depth	***1c9oA***	***1e85A***	***1g9oA_1***	***1g9oA_2***	***complex***	***1eia***	***Average***
0.1	5	46.92	59.37	50.93	45.29	37.90	42.60	47.17
0.5	5	47.07	59.59	50.69	45.45	38.08	43.15	47.34
0.1	15	47.24	59.03	51.42	45.65	38.07	43.35	47.46
0.5	15	46.87	59.04	51.38	45.89	38.38	43.46	47.50

RBED values show that regression tree model significantly reduces base error and keeps predictive power when applied to a protein not present in the training data. Moreover, the variance of RBED values is very small, meaning that the training process yields models that are stable in performance. Furthermore, the RBED values are lower for models tested on *complex*. Recall that the trajectory *complex* was generated for a complex made of a protein and a ligand, while every other trajectory was generated for a single protein. So, it is likely that *complex* contains H-bonds in situations that did not occur in any of the other trajectories. Both deeper trees and larger data fractions tend to improve model accuracy, but the very small gain is not worth the additional model or computation complexity.

### III. Comparison with FIRST-energy model

We've checked whether regression models can predict the stability of H-bonds more accurately than potential energy alone. Table 3 presents the mean RBED value for a model obtained in the first row of Table 2 relative to the base model that is a regression tree built from the same training data using FIRST_energy as the only predictor. FIRST_energy is a modified Mayo potential [5] implemented in FIRST (a protein rigidity analysis software) [7]. Comparison on all 6 data tables show that the more complex models are significantly more accurate than the models based on FIRST_energy alone.

**Table 3:** Mean RBED values of models using single predictor FIRST_energy

***1c9oA***	***1e85A***	***1g9oA_1***	***1g9oA_2***	***complex***	***1eia***
26.36	27.95	22.63	19.63	19.42	5.65

### IV. Identification of least stable H-bonds

Most H-bond occurrences tend to be stable. So, accurately identifying the weakest ones is important if one wishes to predict the possible deformation of a protein [7]. To evaluate how well our models identify the least stable H-bonds occurrences, we first identify the subset *S* of the 10% H-bond occurrences with the smallest measured stability in each test table *T*. Using a regression tree σ obtained in Section II, we sort the H-bond occurrences in *T* in ascending order of predicted stability and we compute the fraction *w* ∈ [0,1] of *S* that is contained in the first 100×u% occurrences in this sorted list, for successive values of *u* ∈ [0,1]. We call the function *w*(*u*) the identification curve of the least stable H-bonds for σ.

**Figure 2:**
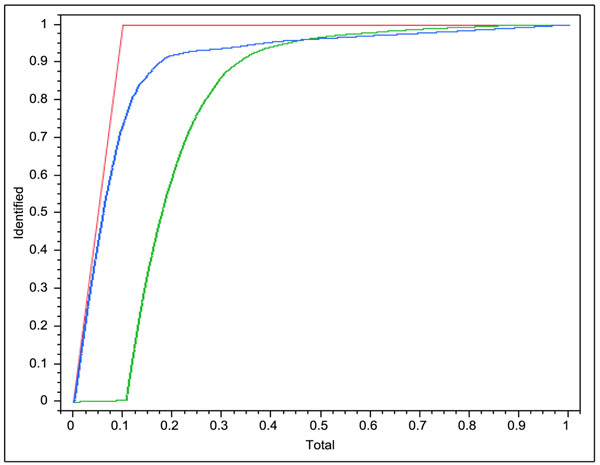
Identification curves of the least stable bonds for *1c9oA* (see Results, Section IV).

Figure 2 plots the identification curve for *1c9oA*. It consists of three curves: the red curve is the (fictitious) ideal identification curve, the blue curve is obtained with one (randomly picked) regression tree computed in Section II, and the green curve is obtained by sorting H-bond occurrences in decreasing values of FIRST_energy. Plots on other proteins present similar curve shapes. For models tested on data tables except *complex*, about 80% of the 10% H-bond occurrences predicted as the least stable are actually among the 10% truly least stable. The results for *complex* are less satisfactory because of the reasons discussed in Section II. The regression models are consistently better than the FIRST_energy-only models, though for *1eia* the difference is small.

## Discussion

In all our regression trees the root split was done with predictor Dist_H_A (the distance between the hydrogen and acceptor atoms), which therefore appear as the single most discriminative attribute to predict H-bond stability. This observation is consistent with previous findings. Levitt [6] found that most stable H-bonds have Dist_H_A less than 2.07Å. Jeffrey and Saenger [14] also suggested that Dist_H_A is a key attribute affecting H-bond stability, with a value less than 2.2Å for moderate to strong H-bonds. Consistent with these previous findings, the split values of the deepest Dist_H_A nodes in all our regression trees are around 2.1Å. This distance was observed in [6] to sometimes fluctuate by up to 3Å in stable H-bonds, due to high-frequency atomic vibration. This observation supports our decision to average predictor values over windows of *l*’ ticks.

Predictor FIRST_energy is often used in splits close to the root. This is not surprising since it is a function of several other pertinent predictors: Dist_H_A, Angle_D_H_A (the angle between the donor, the hydrogen atom, and the acceptor), Angle_H_A_AA (the angle between the hydrogen atom, the acceptor, and the atom covalently-bonded to the acceptor), and the hybridization state of the bond. Some other distance-based predictors (Dist_D_AA, Dist_D_A, Dist_H_D), angle-based predictors and Ch_type (describing whether the donor and acceptor are from main-chain or side-chain) predictor appear often in regression trees, but closer to the leaf nodes. They nevertheless play a significant role in predicting H-bond stability. For example, as shown in Figure 1, if Angle_H_A_AA is at least 105Â°, the stability is very high (about 0.96); otherwise, it drops to 0.71. The preference for larger angle matches well with the well-known linearity of H-bonds [14].

**Figure 3:**
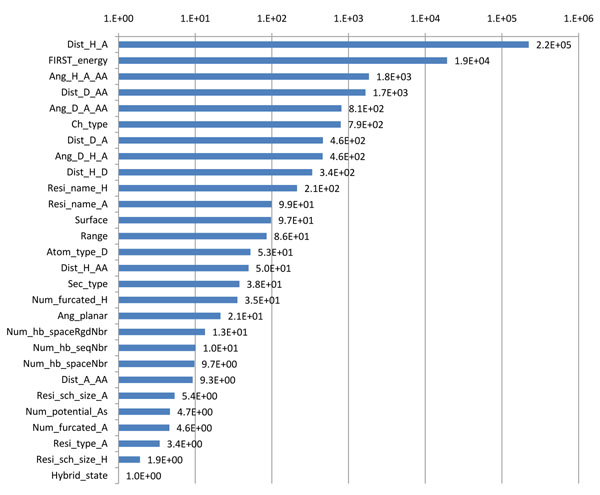
Predictor importance scores

In order to get a more quantitative measure of the relative impact of the predictors on H-bond stability, we define the *importance* of a predictor *p* in a regression tree by: , where *N_p_* is the set of nodes where the split is made using *p*, *w*(*s*) is the score of the split *s*, and *n*(*s*) is the number of H-bond occurrences falling into the node where split *s* is made. We trained 10 models on data tables combining 10% of each of the *6* data tables. Importance scores for each predictor were averaged over these models and then linearly scaled to adjust the score of the least important predictor (with non-zero average importance) equal to 1. The average importance of every predictor appearing in at least one model is shown in Figure 3. The figure confirms that distance-based and angle-based predictors, as well as FIRST_energy, are the most important. It also shows that a number of other predictors—including Resi_name_H, Resi_name_A, and Range (difference in residue numbers of donor and acceptor) —have less, but still significant importance.

Overall, we observe that predictors that describe the local environment of an H-bond play a relatively small role in predicting its stability. In particular, we had expected that descriptors such Num_hb_spaceNbr and Num_hb_spaceRgdNbr, which count the number of other H-bonds located in the neighborhood of the analyzed H-bond, would have had more importance. However, this may reflect the fact that the MD simulation trajectories used in our tests are too short to contain enough information to infer the role of such predictors. Indeed, while transitions between meta-stable states are rare in those trajectories, predictors describing local environments may have greater influence on the stability of H-bonds that must break for such transitions to happen. So, longer trajectories may eventually be needed to better model H-bond stability.

## Conclusions

We have described machine learning methods to train protein-independent regression trees modeling H-bond stability in proteins. Test results demonstrate that trained models can predict H-bond stability quite well. In particular, their performance is significantly better (roughly 20% better) than that of a model based on H-bond energy alone. They can accurately identify a large fraction of the least stable H-bonds in a given conformation. However, our results also suggest that better results could be obtained with a richer set of MD simulation trajectories. In particular, the trajectories used in our experiments might be too short to characterize the stability of H-bonds that break and form during a transition between meta-stable states.

We believe that the training methods could be improved in several ways:

- It would be better to averaging predictor values before sub-sampling MD simulation trajectories. This would reduce the risk of filtering out changes in predictor values that are important for H-bond stability. Unfortunately, in our trajectories we only had access to the data after sub-sampling.

- More sophisticated learning techniques could be used. For example, instead of generating a single tree, we could generate an ensemble of trees, such as Gradient Boosting Trees [16].

- Finally, the notion of stability itself could be refined, for example by distinguishing between the case where an H-bond frequently switches on and off during a prediction window and the case where it rarely switches.

## Authors' contributions

All four authors, IC, PY, MM, and JCL, participated in the formulation of the problem, the design of its solution, and the analysis and the interpretation of the results. PY prepared the experimental data. IC adapted a previously available CART software package and ran the experiments. All authors contributed to the writing of the manuscript, read and approved the final manuscript.

## Competing interests

The authors declare that they have no competing interests.
